# Traumatic Avascular Necrosis of the Capitate Bone in a 23-Year-Old Man: A Case Report

**DOI:** 10.7759/cureus.70052

**Published:** 2024-09-23

**Authors:** Efrain I Paredes, Ines E Baquerizo, Valeria D Reina, Melina G Menoscal

**Affiliations:** 1 Faculty of Health Sciences, Universidad Catolica Santiago de Guayaquil, Guayaquil, ECU; 2 Department of Medicine, Hospital Teodoro Maldonado Carbo, Guayaquil, ECU; 3 Medicine, Hospital de la Policia Nacional Guayaquil No. 2, Guayaquil, ECU

**Keywords:** avn, bone, capitate, trauma, treatment

## Abstract

Avascular necrosis (AVN) of the capitate bone is an infrequent condition, mostly secondary to trauma, that causes persistent pain and immobility of the wrist. The following case report presents a 23-year-old male college student with AVN of the capitate bone type 2, according to the Milliez classification. As a treatment, the patient received an intra-articular injection of methylprednisolone and a wrist stabilizer, resulting in a gradual improvement through conservative treatment and not necessitating surgical intervention. This case underscores the effectiveness of conservative management for early-stage AVN and supports considering non-surgical approaches as a primary strategy in some patients, especially if surgery cannot be done or is not preferred by the patient.

## Introduction

Avascular necrosis (AVN) or osteonecrosis of the capitate bone in the hand is an extremely rare condition, with only 61 cases reported up to 2022 [1] that results from an insufficient supply of blood to the bone, causing the wrist: persistent pain, a cracking sensation during movements, swelling, decreased of mobility, and instability [2]. Usually, osteonecrosis is most common in the hip, humerus, and knee but is rarely seen in smaller bones of the wrist such as the capitate and lunate [3].

The etiology remains unknown but some studies have identified several factors that may contribute to the development of AVN of the capitate bone, reflecting the complex pathogenesis of this condition [4]. These factors include systemic lupus erythematosus, repetitive trauma, sickle cell hemoglobinopathies, medication (such as corticosteroids), Gaucher syndrome (a genetic disorder that can disrupt the normal metabolism of the bone and vascular supply because of an accumulation of lipid-laden cells), and alcohol. Therefore, it can be classified as either idiopathic or traumatic, with the latter being the most common presentation.

For the respective diagnosis, imaging studies play an important role. MRI without contrast medium is the "gold standard" in symptomatic and asymptomatic patients due to its high sensitivity and specificity for visualizing bones and joints [5]. A plain radiograph is the first type of imaging study used not only because we can visualize the dimensions, changes in density, and location of the necrotic area but can also help to exclude other pain causes such as fractures or arthritis [6]. Based on your age, stage of the disease, and the amount of damage, the treatment would be decided. The non-surgical treatment can include the use of non-steroidal anti-inflammatory drugs (NSAIDs), physical therapy, and the immobilization of the wrist. On the other hand, there are more invasive treatments such as bone grafting, core decompression, arthroscopy debridement, wrist arthroplasty, or fusion of the wrist [7].

The following case report presents a traumatic AVN in the capitate bone in a 23-year-old patient, whose treatment was non-surgical through the use of a wrist brace and a corticosteroid injection. Even though it was a more invasive approach with arthroscopic revascularization surgery, through the conservative treatment he received, he steadily improved.

## Case presentation

**Figure 1:**
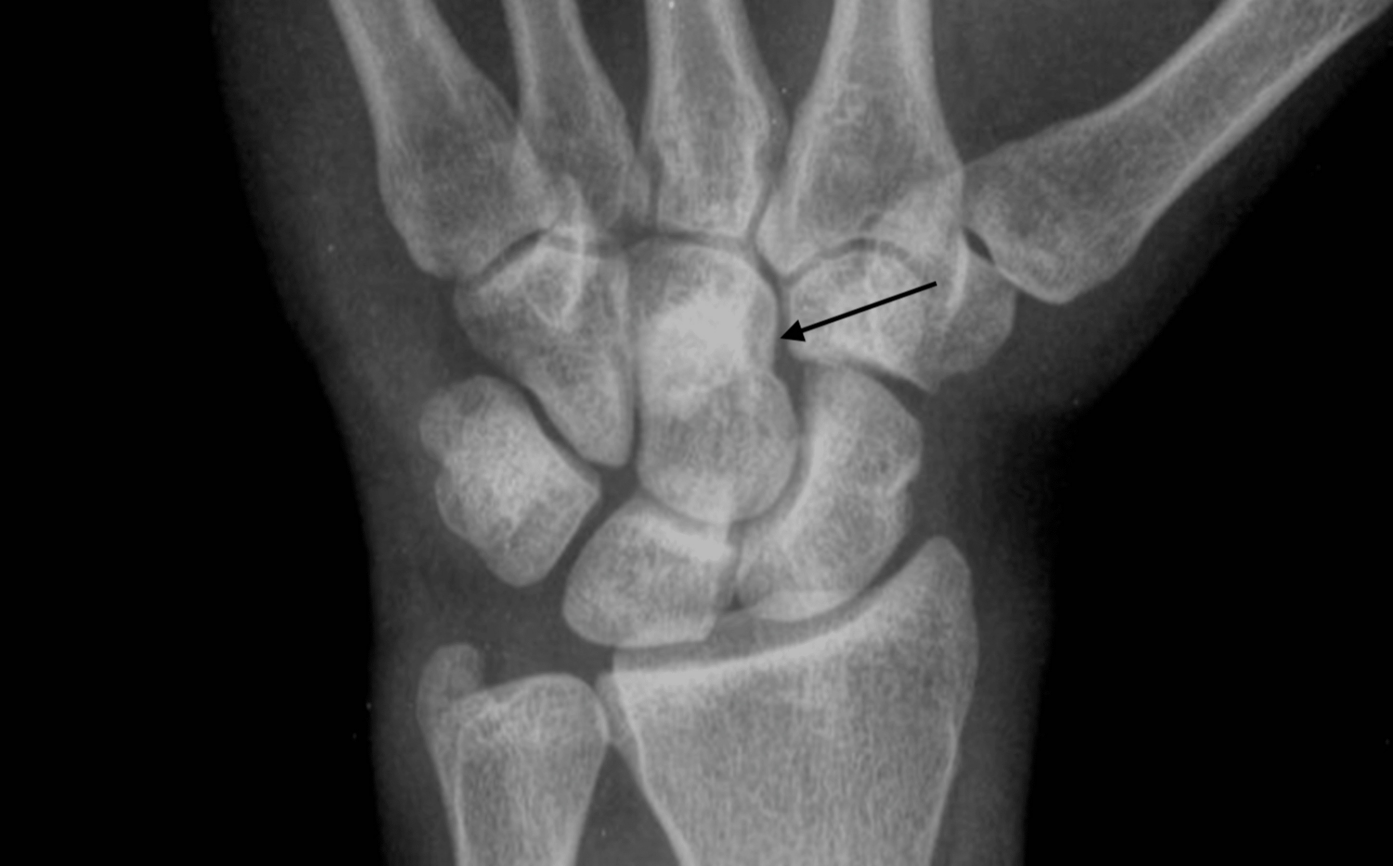
Anteroposterior (AP) right-hand X-ray: opacity in the capitate bone suggesting sclerotic changes (black arrow) (January 2023)

A 23-year-old male college student with no concerning past medical history came to the orthopedic clinic in January of 2023 for pain, soreness, and decreased mobility of the right wrist after hitting a boxing bag without proper protection and gloves. A wrist and hand X-ray were done, as can be seen in Figure 1. He was diagnosed with a wrist sprain and was advised to use a wrist splint for two weeks, to apply ice, and to take meloxicam 15 mg daily for two weeks.

Seven months later, in August of 2023, the patient came to a different orthopedic clinic for a consultation because his pain did not go away and was increasing. He completed his last treatment effectively, and one week after completing the duration of his wrist splint, he returned to the gym. He stated that, initially, he did not have any pain or decreased mobility in the wrist, but after three to four months of doing exercise at the gym, the pain and mobility got worse than it was at the beginning. During the consult, hand inspection, palpation, and range of motion were assessed. Palm pain was measured using the Visual Analogue Scale (VAS) and quantified 90 mm. The range of motion was evaluated through the Watson test, which revealed wrist pain and a 50% reduction in mobility, diminished grasp strength, and inconsistent results. These findings indicate that pain has substantially impaired movement and contributed to the observed functional limitations.

**Figure 2:**
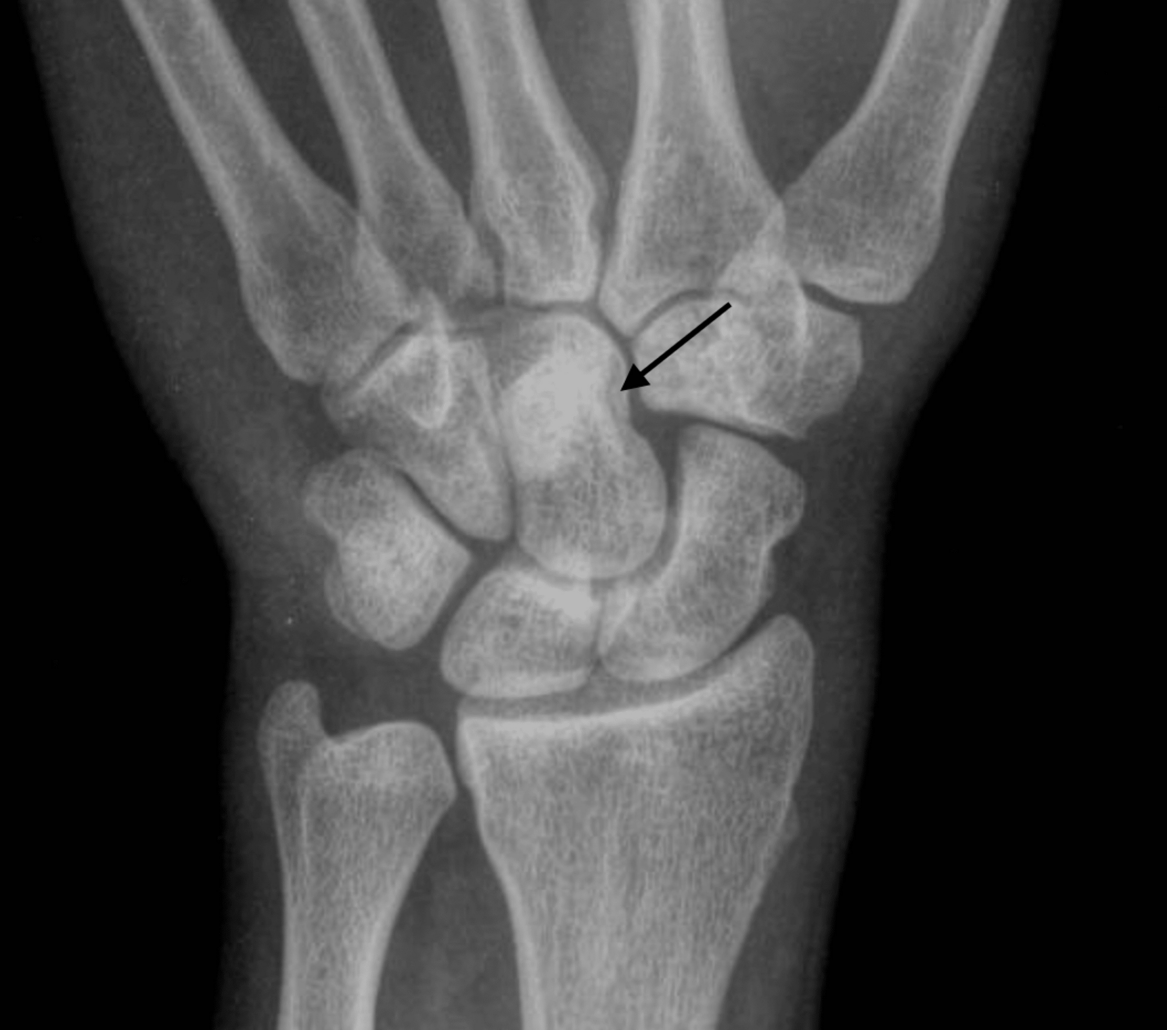
Anteroposterior (AP) right-hand X-ray: opacity in the capitate bone suggesting sclerotic changes (black arrow) (August 2023).

**Figure 3:**
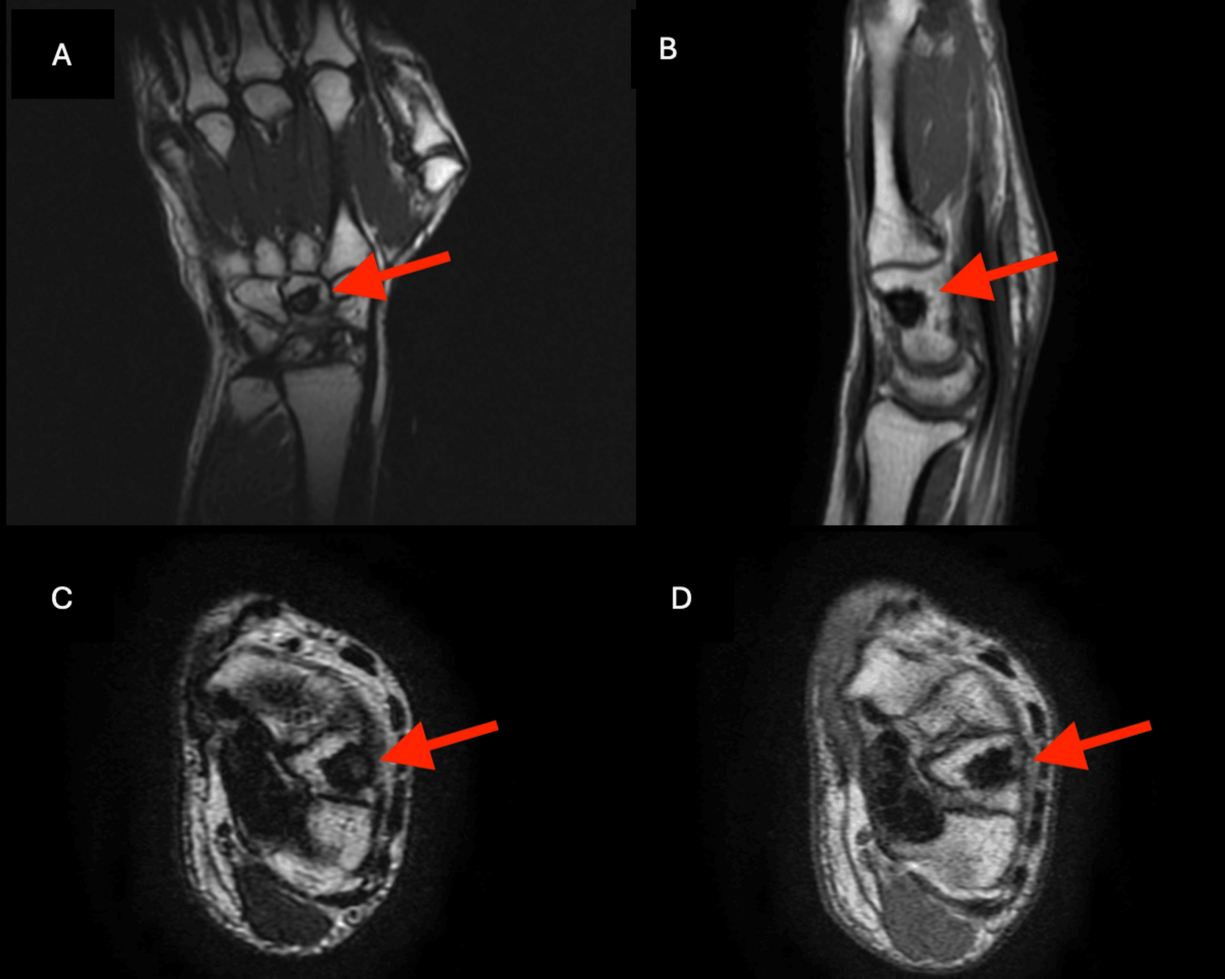
Right-hand MRI revealing a hyperintensity (red arrow): (A) T2-weighted coronal view; (B) T2-weighted sagittal view; (C) T2-weighted axial view; (D) T1-weighted axial view.

**Figure 4:**
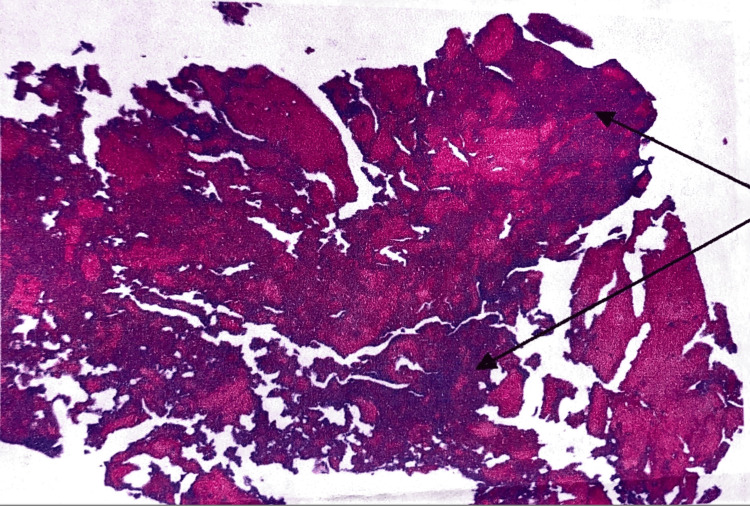
Capitate bone biopsy: fragments of the mature lamellar bone with signs of bone necrosis, little exudation of neutrophils, and blood material (black arrow).

A wrist and hand X-ray were done again to further investigate the cause of the pain. This showed an opacity in the capitate bone of the right hand, as can be seen in Figure 2. Then, an MRI of the hand and wrist was done to further assess and confirm a diagnosis, which showed the findings in Figure 3. All three views, coronal, sagittal, and axial, revealed a hyperintensity in the distal portion of the capitate bone of the right hand, seen in Figures 3A-3D. At the time, four differential diagnoses were suspected, AVN of the bone, infection, a tumor, or a cyst. To confirm the diagnosis, a percutaneous biopsy of the capitate bone was scheduled and performed four days later without any complications. The pathological report evidenced fragments of mature lamellar bone with signs of bone necrosis, little exudation of neutrophils, and blood material, as seen in Figure 4. The patient was then confirmed with a diagnosis of AVN of the capitate bone type 2, according to the Milliez classification.

For the final treatment, a dose of intraarticular methylprednisolone was injected at the level of the first row of the carpal bones to reduce the surrounding inflammation that was causing the pain and decreased mobility, and a wrist splinter was then placed for three weeks. The patient was advised to follow up with a physiotherapist after the wrist splint removal. The patient came to the clinic for a follow-up six months later; his pain and tenderness were gone, which were measured once again with VAS, rating 0 mm; and was able to regain complete wrist mobility according to the Watson test.

Upon contacting the patient in August 2024, 12 months after his final treatment, he reported being pain-free and noted that his wrist functionality remained consistent with the assessment conducted six months prior. He also indicated no difficulties performing physical activities, such as playing tennis or weightlifting at the gym, and confirmed that he had not experienced any recurrence of wrist pain.

## Discussion

AVN of the capitate bone is an extremely rare condition, mainly caused by trauma, that is not very well documented in the recent literature and lacks a clear consensus on its treatment. As of today, there are many treatment options available for this condition. According to the degree of the injury, how much of the blood supply of the capitate bone is lost, the signs and symptoms, and the patient’s preference, age, and comorbidities, non-surgical or surgical treatment can be recommended. Currently, the main surgical options for AVN of the capitate bone are soft tissue interposition and intercarpal arthrodesis, resection of the proximal pole, and revascularization procedures [8]. Although most of the documented cases were treated surgically, less invasive treatments, like the one in this case report, have shown efficacy in a follow-up of 12 months [9]. A study in 2022 that presents two female patients with a similar clinical presentation and age as our case report with capitate AVN type 1 was successfully treated with revascularization of the capitate [8]. Given that most of the successful case reports were treated surgically and are type 1 capitate-AVN according to the Miliez classification, the successful recovery, according to his functional and pain improvement, of this patient’s type 2 capitate-AVN, wrist mobility, and pain with a methylprednisolone infiltration and a wrist splint for three weeks followed by physiotherapy make this case report interesting and suggest further research for a conservative approach for treatment. Another case report in 2022 showed a 27-year-old female patient with traumatic AVN of the capitate bone who initially had a conservative treatment with NSAIDs and a wrist splint for two months, but because there was worsening of the pain and range of motion, an arthroscopic removal of the capitate bone was performed, which successfully improved the patient’s symptoms [10].

Because there are not enough documented cases of this condition, there is not enough evidence to prove that conservative management is significantly successful. Furthermore, because the follow-up in this patient is relatively short, with only 12 months, more case reports with longer follow-up periods need to be done to consider this approach more successful.

Early detection of avascular necrosis is crucial for successful treatment. Since AVN often progresses without obvious symptoms, careful monitoring and diagnosis are essential. Acting quickly in the early stages helps protect joints, reduce pain, and avoid invasive procedures like joint replacement.

## Conclusions

AVN of the capitate bone is an infrequent and under-researched condition that often prompts consideration of surgical interventions, such as capitate revascularization. However, our findings in a 23-year-old patient indicate that a conservative, non-surgical treatment approach, including intra-articular methylprednisolone injection, wrist immobilization with a splint, and subsequent physiotherapy, can lead to significant improvement. This case supports the potential efficacy of non-invasive management in alleviating symptoms and restoring wrist function. Although definitive guidelines for the treatment of capitate AVN are lacking, this case offers strong evidence that conservative management can yield favorable outcomes in the mid-term, even in cases traditionally considered for surgical intervention. The success of non-surgical treatment in this instance underscores the potential for similar approaches in patients with less advanced disease, especially in those who do not want surgery. Thus, conservative therapy can be considered a viable first-line option, with surgical procedures reserved for cases where non-invasive methods prove insufficient. This approach not only minimizes surgical risks but also maximizes the potential for functional recovery with minimal intervention.
